# Healthcare professionals' perspectives on the use of medicinal cannabis to manage chronic pain: A systematic search and narrative review

**DOI:** 10.1111/papr.13161

**Published:** 2022-09-10

**Authors:** Katherine Y. C. Cheng, Joanna E. Harnett, Sharon R. Davis, Daniela Eassey, Susan Law, Lorraine Smith

**Affiliations:** ^1^ School of Pharmacy, Faculty of Medicine and Health The University of Sydney Camperdown New South Wales Australia; ^2^ Institute of Health Policy, Management and Evaluation University of Toronto Toronto Ontario Canada

**Keywords:** chronic pain, healthcare professionals, medicinal cannabis, narrative review, perspectives

## Abstract

**Rationale, aims, and objectives:**

Chronic pain is a global public health problem that negatively impacts individuals' quality of life and imposes a substantial economic burden on societies. The use of medicinal cannabis (MC) is often considered by patients to help manage chronic pain as an alternative or supplement to more conventional treatments, given enabling legalization in a number of countries. However, healthcare professionals involved in providing guidance for patients related to MC are often doing so in the absence of strong evidence and clinical guidelines. Therefore, it is crucial to understand their perspectives regarding the clinical use and relevance of MC for chronic pain. As little is known about attitudes of HCPs with regard to MC use for chronic pain specifically, the aim of this review was to identify and synthesize the published evidence on this topic.

**Methods:**

A systematic search was conducted across six databases: MEDLINE, EMBASE, CINAHL, Scopus, Web of Science, and PubMed from 2001 to March 26, 2021. Three authors independently performed the study selection and data extraction. Thematic analysis was undertaken to identify key themes.

**Results:**

A total of 26 studies were included, involving the United States, Israel, Canada, Australia, Ireland, and Norway, and the perspectives of physicians, nurses, and pharmacists. Seven key themes were identified: MC as a treatment option for chronic pain, and perceived indicated uses; willingness to prescribe MC; legal issues; low perceived knowledge and the need for education; comparative safety of MC versus opioids; addiction and abuse; and perceived adverse effects;

**Conclusion:**

To support best practice in the use of MC for chronic pain, healthcare professionals require education and training, as well as clinical guidelines that provide evidence‐based information about efficacy, safety, and appropriate dosage of products for this indication. Until these gaps are addressed, healthcare professionals will be limited in their capacity to make treatment recommendations about MC for people/patients with chronic pain.

## INTRODUCTION

Chronic pain is a global public health problem that can restrict an individual's physical activity and reduce their quality of life.^1^ It also places a substantial economic burden on individuals, healthcare systems, and societies due to the costs of pharmaceuticals, healthcare, productivity loss, and absenteeism.^2^ Due to the complex nature of chronic pain, the clinical management often involves a range of treatment modalities, including pharmaceutical and psychological treatments, but is often insufficient to provide enough relief.^3^ Thus, many people living with chronic pain have turned to novel and alternative approaches, such as medicinal cannabis (MC), to manage their pain. Ineffective analgesia and a preference for nonopioid treatment are known precipitators for patients to explore the use of MC.^4^ Chronic pain is one of the major reasons for MC use.^5^, ^6^, ^7^, ^8^

Within this context, several countries have introduced legislation in the past two decades to legalize cannabis for therapeutic use. Despite the evident shifts in legislation internationally, there has not been the same progress in building the evidence base of randomized clinical trials to support the use of MC for chronic pain.^9^ Several studies have gathered patient perspectives regarding the benefits of MC for pain control.^10^, ^11^, ^12^, ^13^ While patient perspectives provide important experiential evidence, gaps remain in the scientific evidence related to MC's effectiveness as a pharmacological treatment for chronic pain. Systematic reviews of placebo‐controlled trial data point out the difficulties in comparisons across clinical trials due to, for example, small sample sizes and short duration of studies to measure the outcome of pain control.^14^, ^15^, ^16^, ^17^, ^18^ It was also noted that studies used different MC products which varied in cannabinoid content and formulation, and studied different chronic pain phenotypes, which make it inappropriate to directly compare data. There is a need for high‐quality randomized controlled trials which use a standardized MC product in a defined pain phenotypic population. However, there are unique ethical factors involved in MC research in humans due to its classification as a prohibited drug across much of the world.^19^ In summary, the limitations of the existing evidence coupled with the growth in demand pose a dilemma for healthcare professionals who are caught between patient reports of effectiveness and the absence of high‐quality clinical trials and clinical guidance.

As healthcare professionals are essential for the delivery of healthcare services, their views on the appropriateness of MC use for chronic pain will affect their provision of MC to their patients. Several systematic reviews have been published regarding the effectiveness of MC for different types of pain,^20^, ^21^, ^22^ but these did not elicit the views of the healthcare professionals tasked with prescribing, dispensing, or administering MC.

To date, one systematic review by Gardiner et al.^23^ has been published on healthcare professionals' beliefs, knowledge, and concerns surrounding MC use, however, this considered medicinal use generally and did not focus on chronic pain. The authors concluded that healthcare professionals generally supported MC use in clinical practice but lacked confidence and self‐perceived competence; lacked self‐perceived knowledge about MC in legislative and clinical domains; and held concerns over psychiatric adverse effects and societal harm from recreational misuse of MC.

The aim of our systematic search and narrative review is to build on previous research to identify and synthesize the existing literature on the perspectives of healthcare professionals about the use of MC in the clinical management of chronic pain. For the purposes of this review, MC refers to cannabis that has been prescribed by a healthcare professional for medical purposes and excludes recreational use or self‐medication.

## METHODS

To meet the aims of this study, we adopted a systematic search and narrative review approach to capture publications of interest from a broad range of published studies,^24^ inclusive of study designs and methods, both quantitative and qualitative, that aimed to capture the perspectives of healthcare professionals.

### Search strategy

**FIGURE 1:**
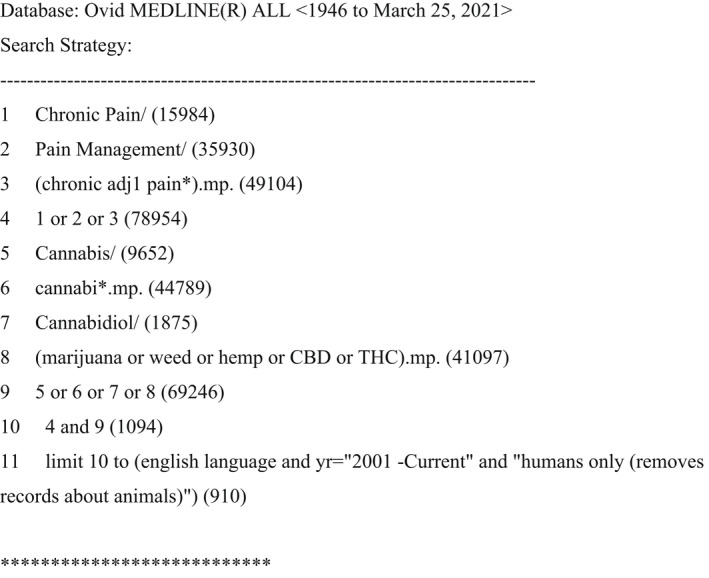
Unique search strategy applied to the MEDLINE database

The following databases were searched: MEDLINE, EMBASE, CINAHL, Scopus, Web of Science, and PubMed. Publications from 2001, the year of the first instance of legalization of cannabis for medicinal use in the world as introduced by Canada, to March 26, 2021 were included. A systematic search of the literature was conducted by creating search strategies for each database, which were modified in accordance with the subject headings and keywords specific to each database. Keywords used were “cannabis,” “marijuana,” “weed,” “hemp,” “CBD,” “THC,” and “chronic pain.” An example search strategy for MEDLINE can be seen in Figure 1. An academic health services librarian was engaged to help refine the search. Additionally, the reference lists of relevant articles were inspected to identify additional publications that were not retrieved via the database search.

### Study screening

**FIGURE 2:**
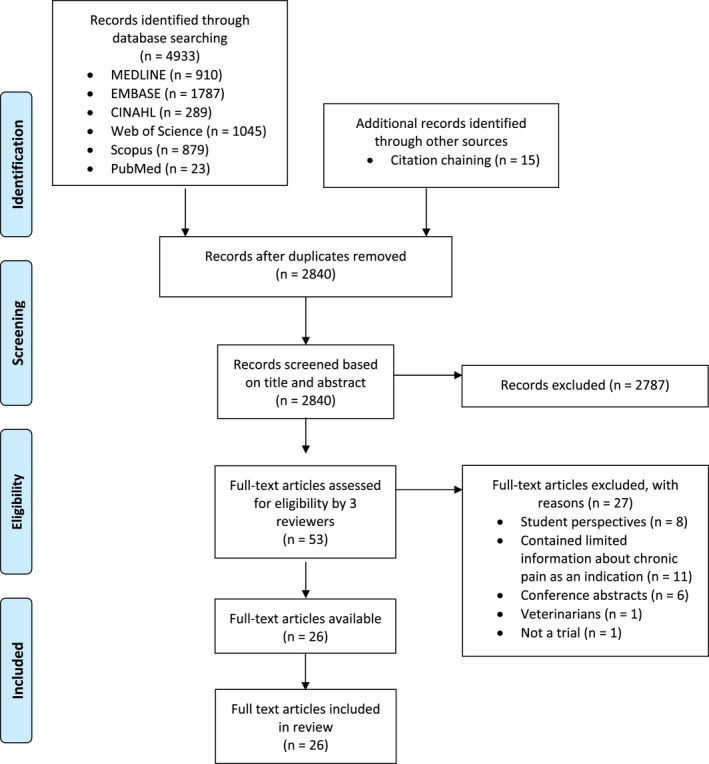
PRISMA flow chart of included and excluded publications regarding the use of medicinal cannabis in chronic pain management.

This systematic search followed the Preferred Reporting Items for Systematic Reviews and Meta‐Analyses (PRISMA) checklist (Figure 2). A screening of titles and abstracts was performed by KC to select potentially relevant publications. A second screening of full‐text articles was conducted by KC, LS, and JH to assess the eligibility for inclusion in the review and resolve any conflicts. Articles which contained limited data on chronic pain were discussed between the three authors to judge their eligibility for inclusion in this review. Inclusion criteria were: healthcare professional participants; related to “medicinal” cannabis; related to the indication of chronic pain; related to perspectives; of English language; human studies; publication date (2001 to March 26, 2021); journal articles or primary studies. Exclusion criteria were: participants did not involve healthcare professionals; related to “recreational” cannabis; contained insufficient information about chronic pain as an indication; non‐English publications; animal studies; reviews, meta‐analyses, gray literature, conference abstracts, editorials, commentaries, books, or book chapters.

### Data analysis

Data analysis was carried out by first organizing the included studies into a table showing authors, publication year, country, aims, study design, sample size, and relevant study findings (Tables 1 and 2). Next, the studies' findings were systematically and thematically analyzed by discussion between the authors to identify common topics and patterns of meaning, as well as negative or deviant cases (viewpoints that deviated from the main body of evidence).^25^

**TABLE 1:** Studies included in this review about chronic noncancer pain

Author (Year), Country	Aims	Study design	Sample	Study findings
Ablin et al. (2016)^37^, Israel	Achieve a cross‐cultural comparison with Canada on rheumatologists' confidence of knowledge of MC	Survey from Fitzcharles et al. (2014)^43^ study	*N* = 23 rheumatology specialists and residents	78% rheumatologists were not confident in writing prescriptions for MC, 48% believed in its therapeutic role for rheumatic diseases, 51% have previously prescribed it. MC would be considered if patients were refractory to conventional treatment by 82.6%
Abo Ziad et al. (2020)^40^, Israel	Assess the attitudes and beliefs of Israeli family physicians on the use of MC and their knowledge on adverse effects and barriers to use	Survey	*N* = 152 physicians (97 specialists, 48 residents, three general practitioners)	High proportions of family physicians believed MC is effective in cancer pain (84.2%) and chronic pain (82.2%). They generally believed specialists in pain medicine and oncology should recommend it. They themselves were *not willing* to prescribe or only *willing to a small extent*
Arnfinsen et al. (2021)^50^, Norway	Assess Norwegian physicians' perceived knowledge of, experience with, and attitudes toward MC	Survey	*N* = 102 physicians	Of the physicians who were asked to choose justifications for why MC should be available by prescription in Norway, the majority stated that MC can improve the quality of life for patients with chronic pain and MC may reduce unnecessary use of opioids
Bega et al. (2017)^51^, International	Assess MC‐related practices, experiences, beliefs, and attitudes of neurologists across National Parkinson's Foundation Center of Excellence	Survey	*N* = 56 neurologists	Physicians described a lack of formal education on MC, reported obtaining their information from medical literature and personal experience, and wanted more attention to MC in the curricula. Concerning side effects noted include negative impact on memory, executive function, driving, and addiction
Carlini et al. (2015)^29^, U.S.A.	Assess Washington State's healthcare providers' MC knowledge, beliefs, clinical practices, and training needs	Survey	*N* = 494 healthcare providers (physicians, physician assistants, nurses, pharmacists)	Intractable pain was the leading indication for written authorizations. Despite healthcare providers' eligibility to write MC authorizations, the majority has not issued one. There was general support for formal training and concerns for addiction and psychiatric side effects
Collister et al. (2021)^45^, Canada	Elicit Canadian nephrologists' views regarding the use and study of MC in patients with kidney disease	Survey	*N* = 154 nephrologists	Chronic pain was one of the major indications for a MC prescription but only 19% of nephrologists have prescribed MC for it. Greater support for MC use in chronic pain if symptoms were refractory to or patients could not tolerate conventional treatment. However, nephrologists did not feel comfortable prescribing MC themselves and preferred someone else do it with more prescribing expertise and a license. Concerns expressed include the lack of evidence for safety and efficacy and uncertainty with dosing
Cooke et al. (2019)^26^, U.S.A.	Present patients' and clinicians' perspectives on the co‐use of MC and opioid among chronic noncancer pain patients with a history of a substance‐use disorder and their clinicians	Semistructured interviews	*N* = 23 primary care providers (18 physicians, four nurse practitioners, one physician assistant)	Clinicians did not routinely discuss MC with patients, but noted that MC was helpful to their patients in managing pain symptoms while on opioids. Clinicians were not comfortable to discuss and/or provide MC in clinical settings and expressed concerns about potential exacerbation of mental health issues with MC use
Crowley et al. (2017)^49^, Ireland	Investigate attitudes of Irish General Practitioners (GPs) attitudes toward decriminalization of MC and assess levels of support for use of MC	Survey	*N* = 565 GPs	>60% Irish GPs agreed that MC has a role in pain management and 58.6% supported legalization for medical use. Support for legalization was higher in those who were trained in addiction treatment (e.g., managing opioid users). Majority of Irish GPs did not support decriminalization of MC for recreational use but GPs with more advanced addiction specialist training supported decriminalization of MC
Ebert et al. (2015)^39^, Israel	Examine the experience, knowledge, and attitudes of Israeli physicians of different specialties toward MC, and assess whether physicians with more knowledge or experience have different attitudes than those who are less knowledgeable and experienced	Survey	*N* = 72 physicians (28 oncology, five pain, 20 psychiatry, 13 neurology, six rehabilitation)	Pain was two of the four most prevalent indications for MC use (chronic pain, cancer pain, chemotherapy‐induced symptoms, palliative care). Oncologists and pain physicians generally disagreed that MC undermined mental health. Physicians who recommended MC reported themselves having greater knowledge. Physicians unanimously agreed that more education on MC should be available to physicians and specific training should precede ability to recommend MC
Fitzcharles et al. (2014)^43^, Canada	Examine rheumatologists' confidence about cannabinoids in general and their ability to advise patients about MC	Survey	*N* = 128 rheumatologists	45% rheumatologists believed there is no therapeutic role for MC in the management of rheumatic diseases, 70% have never previously recommended any form of MC for patients and 60% currently would not recommend a trial of any MC
Irvine et al. (2006)^46^, Australia	Obtain information about GPs' views on potential legalization of MC	Survey	*N* = 32 GPs	GPs generally regarded chronic pain as a MC‐treatable condition. They would prescribe MC if it was legal, backed up by research, and under a legalized regulatory scheme for indications. There was concern of bureaucracy of paperwork with the public knowing about MC access
Jacobs et al. (2019)^48^, Australia	Assess Australia psychiatrists' and psychiatry trainees' knowledge, attitudes, and concerns about MC	Survey	*N* = 23 psychiatrists	>2/3 respondents believed there was evidence for use of CBD and THC in treating chronic pain. They generally could not differentiate between CBD and THC indications. 55% were open to prescribing MC if it were legal. Greatest concerns regarded psychotic symptoms, recreational use (for intoxication), apathy, addiction, and dependence
Karanges et al. (2018)^47^, Australia	Examine the knowledge and attitudes of Australian GPs toward MC	Survey	*N* = 640 GPs	61.5% GPs have fielded at least one enquiry about MC in the last 3 months, but half were not comfortable dealing with enquiries. They believed in the utility of MC, but generally did not want to prescribe MC, citing risk of abuse and dependence as primary concerns. GPs preferred training and accreditation or a shared care arrangement with a specialist instead of a specialist‐only prescribing approach. Most rated themselves having poor perceived knowledge of MC. There was a contrast in support for MC for patients with chronic cancer pain (80.2%) and chronic noncancer pain (39.1%)
Kondrad and Reid (2013)^28^, U.S.A.	Gather information about Colorado family physicians' experience with and attitudes toward MC	Survey	*N* = 520 family physicians	Despite Colorado having the highest per‐capita number of patients using MC, 31% of family physicians never recommended MC. Most physicians did not think there are significant health benefits to MC. Those who have not recommended MC before cited news media, practice policy, personal opinion, concerns of legal liability, and a lack of evidence as influencing factors. Physicians believed MC is predominantly used by people who want legal protection for recreational cannabis. They expressed a strong desire for educational opportunities
Mitchell et al. (2016)^44^, Canada	Determine hospital pharmacists' opinions about MC and their level of comfort in providing advice about MC to patients and other healthcare professionals	Survey	*N* = 769 hospital pharmacists	Majority obtained self‐directed education on MC. Despite limited education, 53.8% of hospital pharmacists have been confronted with questions about its use. 67% disagreed that they were knowledgeable about MC, and 50% completely disagreed that they were comfortable in providing advice to patients
Narouze et al. (2020)^31^, U.S.A.	Characterize pain physicians' advocacy and concerns regarding MC	Survey	*N* = 30 pain physicians	Pain physicians believed in the legitimacy and benefits of MC for physical symptoms and 76% have cared for patients on MC. However, 13% participants were actually registered in an MC program
Philpot et al. (2019)^36^, U.S.A.	Understand the attitudes, beliefs, and knowledge of primary care providers and identify ongoing barriers, biases, and knowledge gaps relating to MC	Survey	*N* = 62 primary care providers (physicians, nurse practitioners, physician assistants)	Providers believed that MC is a legitimate medical therapy for intractable pain but did not believe it can improve patient's quality of life. One third were concerned about significant drug interactions with MC. 50% were not ready or did not want to answer patient questions regarding MC
Sharon et al. (2018)^38^, Israel	Examine the attitudes, beliefs, and personal experiences of Israeli pain specialists regarding the MC	Survey	*N* = 50 pain specialists	95% prescribed MC in their pain practice, 63% found it moderately to highly effective in treating patients with intractable chronic pain, 81% felt that they have not received adequate education regarding MC during their specialty training. There was general agreement that MC has a favorable side effect profile compared to opiates yet most preferred trialing opiate therapy before initiating MC. Doctors favored chronic pain states with a clear etiology and viewed psychiatric morbidity, breastfeeding, and young age to be leading contraindications. Most physicians overestimated addiction rates
Sideris et al. (2018)^30^, U.S.A.	Assess New York's physicians' comfort level, opinions, and experience in recommending or supporting patient use of MC	Survey	*N* = 164 physicians (primary care, anesthesiology, pain medicine, surgery, psychiatry)	87% respondents were not registered with New York's MC program, but majority were willing to recommend patients to registered physicians. Pain symptom control was the most common symptom qualifying for MC. ~75% opioid prescribers considered recommending MC as an adjuvant in pain
Starrels et al. (2020)^27^, U.S.A.	Generate consensus among clinicians expert in chronic pain and opioid prescribing about how to respond to MC use among patients on long‐term opioid therapy (LTOT)	Analysis from an online Delphi study	*N* = 42 clinicians (physicians, nurse practitioners, clinical pharmacologist, registered nurse, clinical nurse specialist)	There was expert consensus about initiating monitoring strategies in response to MC use among patients prescribed LTOT; tapering or discontinuation of LTOT was not important for one‐time or occasional MC users. There was disagreement as to whether to taper or discontinue opioids for patients with a pattern of repeated use and a suspicion of a cannabis use disorder

*Note*: This review uses the standardized abbreviation to encompass “medical cannabis,” “medicinal cannabis,” “medical marijuana,” “cannabis for therapeutic purposes,”, “cannabis,” “cannabinoids,” and “marijuana.”

Abbreviation: MC, medicinal cannabis.

**TABLE 2:** Studies included in this review about chronic cancer pain

Author (Year), Country	Aims	Study design	Sample	Study findings
Braun et al. (2017)^33^, U.S.A.	Investigate the extent to which oncology experts view marijuana as having medical value and the range of approaches to clinical decision making around MC	Semistructured interviews	*N* = 15 oncology experts (10 oncologists, two palliative care physicians, one psychiatrist, one surgeon)	13 of 15 of oncology experts endorsed MC for the indication of pain. Expert opinion was divided on MC's position in medicine with about half who believed MC to have efficacy comparable to conventional management strategies. Nearly as many viewed it as an adjunct, capable of reducing benzodiazepine and opioid loads
Braun et al. (2018)^32^, U.S.A.	Examine beliefs, knowledge, and practices of oncologists regarding MC	Survey	*N* = 237 oncologists	There was a lack of consensus among oncologists regarding MC as the primary treatment for pain, but >2/3 supported its use as an adjunct to standard pain management strategies. 45.9% reported discussing about MC with patients in the past year, 56.7% of them did not consider themselves sufficiently knowledgeable to make MC recommendations
Luba et al. (2018)^34^, U.S.A.	Examine the attitudes, beliefs, and practices of palliative and hospice care providers regarding the use of MC for terminally ill patients	Survey	*N* = 426 palliative and hospice care providers (345 doctors, 58 nurses)	Majority of palliative and hospice care providers saw MC as helpful in treating pain and end of life generally, and useful as an adjuvant medicine. However, fewer than half (46.4%) have recommended MC in the past. 61% indicated that they would recommend MC for terminal illness regardless of legality
Uritsky et al. (2011)^35^, U.S.A.	Assess the knowledge, experience, and views of hospice professionals regarding the use of MC in terminally ill patients	Survey	*N* = 209 hospice professionals (13 medical doctors, 131 nurses, 34 social workers)	90% hospice care professionals supported the legalization of MC for palliative symptoms. Pain control was the most commonly perceived reason for marijuana use though not a currently FDA‐approved indication. Majority would neglect ethical implications with smoking MC if it was controlling symptoms
Zolotov et al. (2018)^41^, Israel	Understand the views of physicians who regularly encounter cancer and chronic pain patients on MC and its possible integration into their clinic, and identify potential underlying factors that influence these perceptions	Semiconstructed interviews	*N* = 24 physicians (six pain medicine, nine oncology, nine family medicine)	On one hand, physicians abiding by the evidence‐base paradigm of healthcare did not see MC as a conventional medicine. Some physicians viewed patients as drug addicts and feared recreational use of medically acquired cannabis. On the other hand, physicians saw MC as a viable treatment for pain and suffering especially for cancer patients
Zolotov et al. (2019)^42^, Israel	Identify underlying factors that influence physicians' intentions to recommend MC to patients	Survey and some interviews	*N* = 247 physicians (98 family physicians, 80 oncologists, 69 pain medicine)	Respondents had higher intentions to recommend MC to the cancer patient than the chronic pain patient. There were more psychosocial (nonmedical) factors associated with intentions to recommend MC to the chronic pain patient

*Note*: This review uses the standardized abbreviation to encompass “medical cannabis,” “medicinal cannabis,” “medical marijuana,” “cannabis for therapeutic purposes,” “cannabis,” “cannabinoids,” and “marijuana.”

Abbreviation: MC, medicinal cannabis.

## RESULTS

### Study selection and characteristics

**FIGURE 3:**
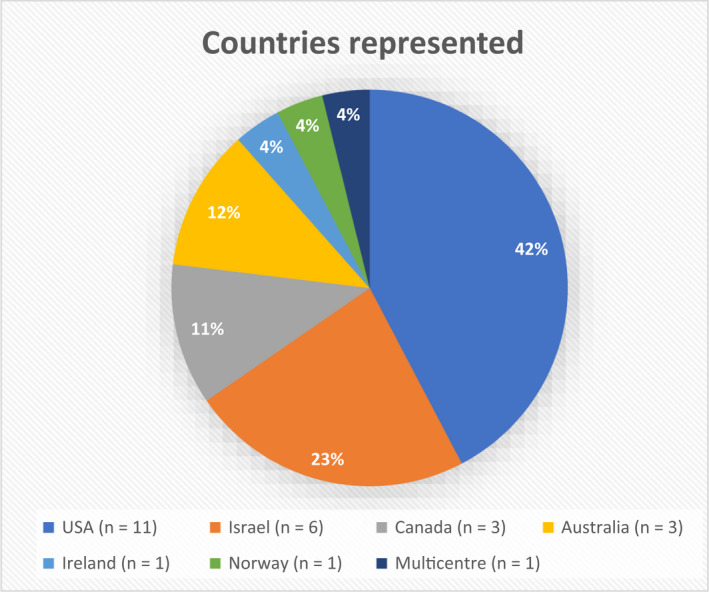
Countries represented in this systematic search and narrative review

**FIGURE 4:**
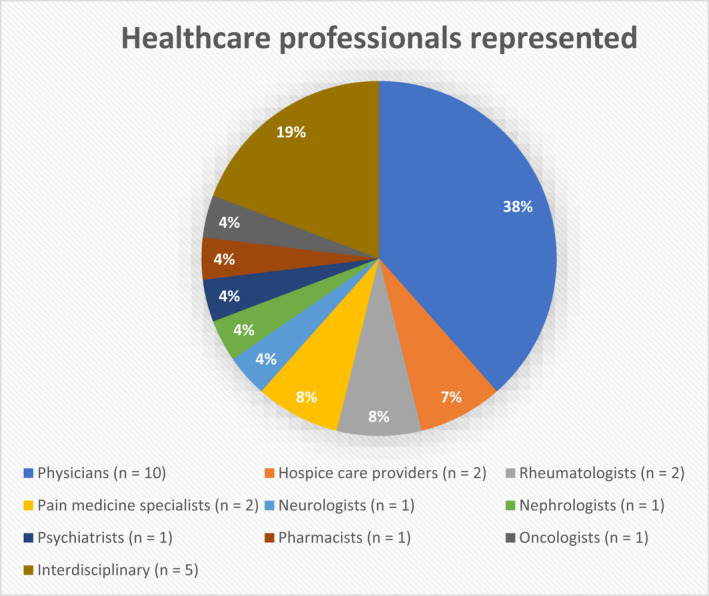
Types of healthcare professionals represented in this systematic search and narrative review. “Interdisciplinary” combines the studies which used a mixed cohort of healthcare providers.

As shown in the PRISMA flow chart (Figure 2), the database search yielded 4933 records. Following the removal of duplicates, 2840 records were screened by title and abstract, resulting in 53 full‐text articles being further assessed against our inclusion and exclusion criteria. A total of 26 studies met the criteria for inclusion (Tables 1 and 2). As shown in Figures 3 and 4, the 26 articles included studies from the United States of America,^26^, ^27^, ^28^, ^29^, ^30^, ^31^, ^32^, ^33^, ^34^, ^35^, ^36^ Israel,^37^, ^38^, ^39^, ^40^, ^41^, ^42^ Canada,^43^, ^44^, ^45^ Australia,^46^, ^47^, ^48^ Ireland,^49^ and Norway.^50^ One study was conducted across five countries.^51^ Healthcare professionals represented in the studies were predominantly physicians,^28^, ^30^, ^39^, ^40^, ^41^, ^42^, ^46^, ^47^, ^49^, ^50^ but also palliative care providers,^34^, ^35^ rheumatologists,^37^, ^43^ pain medicine specialists,^31^, ^38^ neurologists,^51^ nephrologists,^45^ psychiatrists,^48^ pharmacists,^44^ and oncologists.^32^ Some studies included a mixed cohort of healthcare providers,^26^, ^27^, ^29^, ^33^, ^36^ including physicians, physician assistants, osteopathic physicians, osteopathic physician assistants, naturopathic physicians, advanced registered nurse practitioners, registered nurses, licensed nurses, pharmacists, oncologists, palliative care physicians, a psychiatrist, a surgeon, and a clinical nurse specialist. Methods for data collection included surveys,^28^, ^29^, ^30^, ^31^, ^32^, ^34^, ^35^, ^36^, ^37^, ^38^, ^39^, ^40^, ^42^, ^43^, ^44^, ^45^, ^46^, ^47^, ^48^, ^49^, ^50^, ^51^ semistructured interviews asking open‐ended questions,^26^, ^33^, ^41^, ^42^ and a Delphi study analysis.^27^ The sample sizes of studies ranged from 15 to 749 healthcare professionals.

### Thematic analysis

The study findings are presented under the following seven themes: MC as a treatment option for chronic pain and perceived indicated uses; willingness to prescribe MC for patients with chronic pain; legal issues affecting use of MC in patients with chronic pain; low perceived knowledge in and the need for education for healthcare professionals supplying MC for chronic pain; comparative safety of MC versus opioids; potential for addiction and abuse in patients with chronic pain; perceived adverse effects.

#### Medicinal cannabis as a treatment option for chronic pain

The majority of healthcare professionals recognized that MC is a legitimate treatment option for the indication of either chronic pain,^26^, ^27^, ^28^, ^30^, ^31^, ^32^, ^33^, ^34^, ^35^, ^38^, ^39^, ^40^, ^41^, ^42^, ^44^, ^45^, ^46^, ^48^, ^49^, ^51^ intractable pain (pain that is refractory to conventional treatments),^29^, ^36^ or rheumatic diseases,^37^, ^50^ while one study of rheumatologists showed that they generally did not believe that MC has a role in therapeutic management in their practice.^43^ Some healthcare professionals indicated more support for MC use in chronic cancer pain^36^, ^40^, ^41^, ^47^, ^50^ or palliative care^41^, ^46^ than general chronic pain. There were mixed views regarding whether MC improves a patient's quality of life.^36^, ^50^

Perspectives on MC as a treatment option were influenced by the lack of quality control testing of MC products on the market, the particular formulations of MC products available to the healthcare professional, and the healthcare professional's clinical training and experience. Of the 15 oncology experts in one study, almost half expressed reservations regarding the efficacy of MC due to the varied constituents and content between MC products on the market which can result in variations in pharmacological effects and therefore unpredictable therapeutic outcomes.^33^ In terms of the formulation of the products, some Israeli physicians considered smoking as an inappropriate delivery mechanism as the dose delivered cannot be ascertained and the act of smoking is associated with morbidities.^41^ Senior healthcare professionals in another study indicated they would choose to neglect the ethical implications and potential health consequences relating to the act of smoking a medicine if it was controlling the patient's symptoms.^35^ Healthcare professionals who showed a greater endorsement of MC as a treatment option were those who had additional addiction training, and they also showed a greater support for cannabis to be decriminalized for recreational use.^49^ MC as a treatment option was also endorsed by healthcare professionals who had greater accumulated clinical experience with MC. In an Israeli interview, an oncologist said, “I think that we see in the clinic much more efficacy of cannabis than what has been proven in the literature.”^41^ Clinical experience was regarded significantly more influential by physicians who had recommended MC in the past compared to those who had not.^39^ Healthcare professionals who had recommended MC were generally more likely to be convinced of its benefits and less concerned of its risks^28^, ^29^, ^38^ or had greater perceived knowledge of MC.^44^

Physicians' perspectives on appropriate indications for initiating MC were varied. In a Norwegian study, chronic pain was ranked lower as an indication than cancer, multiple sclerosis, rheumatic disease, and Parkinson's disease.^50^ Likewise, few Australian general practitioners supported the use of MC in chronic noncancer pain.^47^ Pain medicine specialists in Israel judged that neuropathic pain, oncological pain, and arthralgias related to rheumatic diseases were the most suitable indications to start MC, with the least suitable being chronic low back pain and chronic postoperative pain.^38^ Almost half (47%) of this sample of 50 pain specialists considered a lack of a clear diagnosis as a contraindication. Israeli family physicians indicated during interviews that it was easier for them to recommend MC to cancer patients because the diagnosis is confirmed, whereas reports of other pain are more subjective.^41^, ^42^

Few studies have investigated the use of MC for end‐of‐life care. Israeli physicians indicated they were not concerned by the lack of sufficient scientific evidence supporting MC and the potential harms of use (eg, addiction, side effects) as long as it helps the suffering patient.^35^, ^41^ Oncology experts regarded those at end of life requiring palliation as the population in which it is most appropriate to use MC.^33^

#### Willingness to prescribe MC for chronic pain

Willingness to prescribe MC among physicians varied. There were higher prescribing rates reported via pain specialists, and rheumatologists from Israel,^37^, ^38^ but most Israeli family physicians were *not at all* willing or only willing *to a small extent* to prescribe MC.^40^ As most American physicians were not registered with the state's MC program – the only way can they legally recommend MC to patients^30^, ^36^ – they preferred to refer patients to another physician or specialist with MC prescribing expertise and licensure,^40^, ^45^ or to prescribe after consulting a specialist.^43^, ^46^, ^47^ Barriers to prescribing included: a lack of evidence for use^28^, ^29^, ^30^, ^34^, ^41^, ^44^, ^45^, ^46^, ^50^; concerns of abuse^40^, ^41^, ^43^, ^50^; bureaucracy involved with getting approval^30^, ^36^, ^40^, ^46^; lack of training and knowledge^29^, ^44^; lack of algorithms or endorsed clinical guidelines^29^, ^41^; federal status of cannabis or political resistance^29^, ^30^, ^33^, ^50^; legal liability or licensure.^28^, ^30^ Rheumatologists were willing to consider a trial period for MC if patients had failed conventional treatments, but were less willing if patients requested the substance.^37^, ^43^

#### Legal issues affecting prescribing of MC for chronic pain

The legal boundaries governing access to MC affected its actual and perceived prescribing for chronic pain. Access varied from country to country, for example, in the United States, cannabis is a prohibited (Schedule I [controlled]) substance at a federal level according to the Controlled Substances Act (1970); however, individual states legally permit the use of cannabis for medicinal purposes if a registered healthcare professional certifies a user's eligibility. Federal and state differences in the legal status of MC were a primary barrier for New York physicians to register into the state program to certify patients for use.^30^ Washington healthcare providers' (including physicians, nurses, and pharmacists) reported a lack of comfort when recommending MC, and expressed that they should not have to fear legal action when allowing a patient to access use.^29^ Hospice care providers and neurologists agreed that MC should be reclassified as a controlled substance in order to change their views on its utility in palliative care and Parkinson's disease, respectively.^34^, ^51^ However, almost half of the surveyed Colorado family physicians (*n* = 520) were not in favor of decriminalizing cannabis and would rather it remain as a controlled drug.^28^ In Norway, MC was legalized in 2016 but has not been readily available for patients due to the strictly regulated approval process and the small number of physicians who can prescribe MC. Norwegian physicians supporting MC on prescription believe that the current legislation prevents optimal quality of care where MC could improve the quality of life for patients with chronic pain.^50^ An Australian study published in 2006 found that 75% of rural general practitioners (*n* = 32) felt prepared to prescribe MC with the only barrier being its status as an illegal drug.^46^

#### Low perceived knowledge and the need for education

Generally, across all disciplines, healthcare professionals involved in managing patients with pain reported being not ready or comfortable to answer patients' questions regarding MC,^36^, ^37^, ^43^ discuss MC with patients or other healthcare professionals,^26^, ^44^, ^47^ write a prescription for MC,^40^, ^43^, ^45^, ^47^ or issue a written authorization for a patient to possess MC.^29^ Related to this finding, few healthcare professionals received formal training or MC‐related education during their undergraduate degree. A total of 48% of Australian general practitioners (*n* = 640) rated their knowledge of MC as poor.^47^ Of Israeli pain specialists (*n* = 50), 81% did not receive adequate education during specialty training.^38^ Insufficient knowledge played into the self‐perceived low ability of oncologists to make a recommendation for MC use.^32^ The majority of healthcare professionals engaged in self‐directed education^44^ with sources of information being medical literature,^29^, ^50^, ^51^ clinical experience,^29^, ^51^ news and media,^29^, ^50^ and colleagues.^29^, ^50^ Despite such patterns of use of information sources, more than 70% of doctors (*n* = 25) perceived that clinical practice guidelines served as a better educational format than peer‐reviewed literature.^48^ There was almost unanimous endorsement of the need to pay more attention to MC in undergraduate curricula.^28^, ^29^, ^51^ Many believed that formal training or a licensing procedure should precede the ability to authorize use for a patient.^28^, ^29^, ^39^, ^47^ A large proportion were interested in learning more about the topic or wanted to be trained to prescribe MC.^36^, ^39^, ^46^, ^47^

#### Comparative safety of MC versus opioids

Opioids have long been the mainstay in the treatment of refractory pain. The general view held by healthcare professionals in this review, for example, by Israeli physicians,^38^, ^41^ Australian general practitioners,^47^ and American oncologists^32^ is that MC is safer than opioids. MC was observed to have the advantages of being opioid sparing,^33^, ^41^, ^50^ having less side effects,^41^ and carrying lower risks of overdose death and addiction.^32^ However, MC was also recognized to cause paranoia and confusion which opioids did not.^32^ There was support for MC to be used as an adjunct to conventional chronic pain management strategies by New York physicians,^30^ a mixed cohort of healthcare providers in Norway (mainly oncologists)^47^ and Israeli oncology experts.^32^ However, when asked about cannabis in practice, a majority of Israeli pain specialists thought that opioid therapy should be trialed prior to commencing cannabis.^38^ A large proportion of the pain specialists preferred themselves and their families to be treated with opioids when given the choice against cannabis if a situation necessitated it.^38^ Also, the majority of American hospice providers in one study (mainly physicians and nurses) was *not sure* or believed that MC was *not as effective* than conventional pain treatment.^34^ Clinicians did not discuss MC with patients on long‐term opioid therapy for pain nor routinely perform urine toxicology testing due to the assumption that patients were using cannabis already, given the prevalent use of cannabis in the chronic pain patient population.^26^

#### Addiction and abuse

Many healthcare providers held the view that cannabis was addictive.^29^, ^35^, ^47^, ^48^, ^51^ This influenced some Australian general practitioners' decisions in one study (about 30% of those sampled; *n* = 640) to not prescribe MC.^47^ Addiction concerns have manifested in physicians' fears that MC for recreational use would be disguised under legal protection.^28^, ^41^ There were unclear boundaries between recreational and medicinal use of cannabis reported by Israeli physicians where they related the therapeutic effect to “getting high.”^41^ Australian rural doctors also feared burdening the healthcare system if patients sought prescriptions for recreational use once MC is legal.^46^

As noted earlier, “when dealing with very sick patients, the lack of evidence and potential harms carried less weight” compared to relief of suffering.^41^

#### Perceived adverse effects

Mental health risks including anxiety, psychosis, paranoia, hallucinations, and dysphoria were commonly cited adverse effects across studies.^28^, ^29^, ^33^, ^39^, ^47^, ^48^, ^50^ One study highlighted the difficulty healthcare professionals had in distinguishing between cannabis‐induced psychosis and a patient's underlying psychiatric disorder.^26^ This may be why Israeli pain specialists noted schizophrenia and previous psychosis as the leading contraindications to commencing MC.^38^ Driving impairment^47^, ^51^ and cognitive impairment^47^, ^51^ were also noted as adverse effects of MC.

## DISCUSSION

There is wide support among healthcare professionals for the inclusion of MC in the management of chronic pain.^33^, ^34^, ^35^, ^36^, ^38^, ^39^, ^40^, ^41^, ^44^, ^45^, ^46^, ^50^, ^51^ Furthermore, chronic pain is the most common reason for authorization or recommendation of MC use.^28^, ^29^, ^30^, ^45^ Indeed our review found that healthcare professionals were frequently presented with inquiries,^30^, ^44^, ^47^, ^50^ requests to prescribe,^41^, ^51^ opportunities to recommend MC,^28^, ^31^ or were confronted with patients in their care who were already taking MC.^31^, ^45^, ^50^, ^51^ However, studies have suggested relatively low prescribing and recommending rates overall, complicated by legal issues, personal perspectives, a lack of education and formal training, and the absence of evidence‐based guidelines for healthcare professionals.

Since the legal use of the cannabis plant was prohibited globally in 1961, access to MC has been complex internationally despite prior traditional use. For some countries, the plant has remained prohibited for both medicinal and recreational purposes, whereas in others unique legal frameworks have been established to uphold the use of MC and/or recreational cannabis.^52^ A variety of access models and regulatory experiences have emerged internationally. The latter occur in the United States where cannabis is illicit at the federal level but prescribers can be registered in state‐based programs to write a “recommendation” for the plant for a patient if the patient suffers from one of the conditions listed by the state.^53^ It remains unclear why healthcare professionals in the United States fear legal action despite being covered by state policy, and this situation presents a barrier to prescribing. While the majority of literature reporting “regulatory experiences” associated with MC in our review is based in the United States, it is important to note such experiences will vary throughout the world. However, international research for MC is certainly hampered by the legalities of accessing it.^22^

This review found a disconnect between healthcare professionals' views and actual practice in the treatment of chronic pain, in that MC was regarded as safer than opioids yet was not prescribed to the same extent as opioids. To date there is little evidence regarding interactions between the use of opioids and cannabis/cannabinoids concurrently.^54^ Vaporized cannabis may reduce pain in chronic pain patients taking morphine or oxycodone without affecting the pharmacokinetics of these opioids. Additive sedation and CNS depression might occur with concurrent use of cannabis or cannabinoids with opioids.^54^

In the last two decades, the world has seen an epidemic of opioid prescribing whereby prescription opioids have been associated with a large number of deaths and labeled a “public health problem.”^55^ Healthcare professionals' positive views regarding cannabis' comparative risk profile to opioids may be shaped by the lives lost to opioid toxicity, such that not using opioids at all or reducing the opioid load are seen as favorable. However, our review saw reservations for MC to be used in practice expressed by Israeli pain specialists, American hospice providers, and American pain experts. This may be attributed to a recent shift toward opioid stewardship and deprescribing in practice, which has consequently seen the emergence of evidence‐based guidelines from public health agencies to address harms from the misuse of these pain medicines.^56^, ^57^ These guidelines serve as a means of dissemination of best clinical practices to prescribers.^58^ One systematic review of 15 studies showed low to moderate evidence that opioid stewardship efforts have decreased the number of opioid prescriptions, number of patients on long‐term opioids, duration of prescribed treatment, and opioid dosages.^59^ Until there are high‐quality randomized controlled trials for MC to test its efficacy and safety for the indication of chronic pain, and standardize the dose and administration for prescribing, local prescribing protocols cannot be evidence based. Meanwhile opioids appear to be the preferred treatment by physicians due to the presence of guidelines. This highlights the need for more funding and attention by regulatory authorities to increase the pace of research approval for MC and trial result turnover, which can be subsequently translated into evidence‐based guidelines to support the healthcare professional after the point of MC's legalization.

Health professionals in our review showed a preference for prescribing MC to patients with chronic pain associated with a clear etiology (eg, neuropathic pain, cancer‐related pain, rheumatic diseases), where the diagnosis is confirmed, as well as those at their end of life. The indication with the most robust evidence for MC is neuropathic pain with a number of double‐blind placebo‐control trials conducted.^17^ However, there remains a lack of evidence for other pain phenotypes.^19^ As there is no objective marker for diagnosis with chronic pain, diagnosis is reliant on the patient's report of their lived experience. This subjectivity of a chronic pain diagnosis may form the basis of physicians' concerns of abuse where they may perceive patients to be malingering under the indication of chronic pain in order to gain access to MC for recreational use. This review found lower intentions to prescribe MC for general chronic pain than in Gardiner et al.’s review,^23^ but the latter was not focused on health professionals practicing in the sphere of chronic pain. There also appeared to be a compassionate stance toward the suffering patients experience in our study, causing physicians to be more permissive with palliative medications and overlook issues of addiction, side effects, and the reluctance to prescribe MC. Interestingly, there are few studies conducted on end‐of‐life care, yet it is the population that is considered the most appropriate to use MC. Perspectives on MC use in end‐of‐life care may therefore be a promising area of research.

While the prescription or approval of MC may currently be restricted to physicians, pharmacists and nurses also have roles in educating their prescribing colleagues, counseling patients about MC's safety and efficacy, staying informed about and interpreting emerging research, as well as dispensing or administering MC, respectively. Additionally, in some parts of the world, pharmacies serve as points of sale of MC (eg, Australia).^52^ Understanding healthcare professionals' personal views regarding the efficacy of MC is crucial as these play into their provision of a perceived viable treatment option for chronic pain. Therefore, the views of other healthcare professionals regarding chronic pain and pain with underlying conditions could be a future research direction.

Finally, it is concerning that few healthcare professionals feel equipped to deal with people who use MC for chronic pain, due to limited opportunities for education and training around MC worldwide. This may be explainable due to the lack of supporting evidence for its efficacy. With the legalization of MC, healthcare professionals often enter practice without prior knowledge or guidance about the substance which has grown in public favor. Consequently, a high use of nonpeer‐reviewed information sources was reported by healthcare professionals in this review. It is encouraging that healthcare professionals expressed a strong desire for formal educational opportunities, which may suggest a willingness to undergo additional training. This review showed that those with more clinical experience have more permissive attitudes to prescribing or recommending MC for chronic pain. While scientific evidence accumulates to inform practice guidelines and educational curricula, healthcare professionals rely on their accumulated clinical experience to guide their present practice. This reinforces the need for safety and efficacy data to be gathered and disseminated, and clinical dosing guidelines to be developed to support the healthcare professional after the point of legalization and ensure the provision of MC to chronic pain patients where appropriate.

### Limitations

First, we did not assess the quality of study methodologies included in this review, given the focus on the narrative and thematic synthesis of the included studies.^24^ Second, we generalized all cannabis‐containing products mentioned in studies under the term “medicinal cannabis,” though perspectives may differ, for example, toward the cannabis plant versus manufactured cannabis products. Third, this review included articles of the English language only. Potential literature may have been missed from countries which are leading in the MC field, but whose scientists may not be publishing results in English. Additionally, this review reported on studies that largely consisted of physicians and specialists involved in the prescribing or recommendation of MC. Future studies could consider a deeper examination of the perspectives of pharmacists and nurses, who may be directly involved in the dispensing or administration of MC, respectively, regarding chronic pain versus pain associated with underlying conditions.

## CONCLUSIONS

Healthcare professionals appear to rely on their clinical experiences of caring for people living with chronic pain to make decisions about MC use. Healthcare professionals require education and training, and clinical guidelines that provide evidence‐based information about efficacy and safety, and guidance related to dosage of MC products for chronic pain. Until these gaps are addressed, healthcare professionals are limited in making informed treatment recommendations about MC, may deny a potentially beneficial intervention, divert patients to inappropriate or unnecessary care, and may be unintentionally devaluing the lived experience and preferences of people with chronic pain.

## AUTHOR CONTRIBUTIONS

All authors have made a significant contribution to this manuscript. LS, JH, and KC conceived the study, collected the data, performed data analysis, and drafted the manuscript. Critical revision of the manuscript was conducted by SD, DE, and SL.

## CONFLICT OF INTEREST

The authors do not have any conflicts of interest to declare.

## Data Availability

The data that support the findings of this study are available from the corresponding author, [author initials], upon reasonable request.
